# Polyphenol Stability and Physical Characteristics of Sweetened Dried Cranberries †

**DOI:** 10.3390/foods9050551

**Published:** 2020-05-01

**Authors:** Kara Kovacev, Brianna Hughes, J. Scott Smith

**Affiliations:** 1 Ocean Spray Cranberries Inc., 1 Ocean Spray Drive, Lakeville, MA 02349, USA; kara.kovacev@yahoo.com; 2 The Town Dock, 45 State Street, Narragansett, RI 02882, USA; bhughes@towndock.com; 3 Food Science Institute, Kansas State University, Manhattan, KS 66506, USA

**Keywords:** proanthocyanidins, anthocyanins, cranberries

## Abstract

There is little research on how product matrix and processing affect phenolic compounds in sweetened dried cranberries over time. The objective of this research was to assess polyphenol content and stability in sweetened dried cranberries between product matrix types. This research assessed five commercially available sweetened dried cranberry matrices: (1) sliced apple juice infused, (2) whole apple juice infused, (3) sliced sucrose infused, (4) whole sucrose infused, and (5) sliced soluble corn fiber, glycerin, sucrose, and sucralose infused (three replicates/treatment). Proanthocyanidins, anthocyanins (HPLC), total phenolic content (Folin–Ciocalteu), water activity, moisture content, color, and texture were evaluated over 12 months at 21 °C. Data were analyzed by ANOVA (*p* < 0.05). Results demonstrate that sweetened dried cranberry polyphenols are unstable regardless of product matrix. More research is needed to determine optimal processing parameters for sweetened dried cranberries to maintain polyphenol stability as healthier food options for consumers.

## 1. Introduction

Functional foods were first introduced in Japan in the 1980s [1], and despite the United States not specifically recognizing functional foods as a legal category, consumers are looking to bring functionality and health to their lives through what they eat. Functional foods can range from whole foods, such as fruits, to those foods that are fortified or enriched with micronutrients, such as vitamins and minerals, or macronutrients such as protein or fiber [2]. When asked about foods that are functional, most consumers look at fruits and vegetables being important and top-of-mind functional foods [3]. Fruits are rich in bioactive compounds such as flavonoids in berries and resveratrol in grapes [4]. Cranberries (*Vaccinium macrocarpon*) are functional fruits composed of bioactive compounds such as anthocyanins and proanthocyanidins, which, in conjunction with other flavan-3-ols, acids, and flavonols make up the overall phenolic content [5].

Anthocyanins are a class of flavonoid which give pigmentation to fruits and vegetables, usually in the form of reds, blues, purples, and blacks [6]. Proanthocyanidins are a part of the flavonoid group also known as condensed tannins. They are polymers of flavan-3-ols and are commonly consumed parts of the human diet [7,8]. 

Fresh cranberries are rich in bioactive compounds, containing 60.42 mg/100 g combined cyanidin and peonidin anthocyanins [9] and 354.9 mg/100 g proanthocyanidins [10]. While cranberries possess well-established health benefits such as promoting urinary tract health [11] and aiding in the prevention of cardiovascular diseases [12], consumers do not generally eat cranberries raw, but instead consume them primarily processed into juices, sweetened dried cranberries, sauces, or supplements [13]. During processing of these products, the cranberries are subjected to high heat which can cause degradation of bioactive compounds. Thermal processing of blueberry purees and juice decreased anthocyanin content by 28–59% [14]. In cranberry juice processing, blanching of the fruit prior to juicing also resulted in a 58–61% reduction in total anthocyanin content compared to a frozen control [15]. There is research quantifying and looking at the polyphenols in cranberry juice drinks [13,16], but many do not cover the stability of these compounds over time, and there is no research looking at these compounds in products such as sweetened dried cranberries. The differentiation on the impact of processing on polyphenol stability between the dried cranberry process and the juice making process is an avenue of research that should be further explored. The dried fruit market is expected to increase 5.7% between 2018–2026 from a combination of convenience and healthier food choices [17]. Sweetened dried cranberries are a convenient snacking option; however, there is a lack of research on polyphenols in sweetened dried cranberries and most of the literature on cranberry polyphenols and processing is focused on juice fractionation and processing. This research evaluated how processing cranberries into sweetened dried cranberries affects the bioactive compounds that cranberries are known for, as well as how stable those compounds are over time.

## 2. Materials and Methods

The study evaluated sweetened dried cranberries (SDC) across 5 treatments (sliced (SAJ) and whole (WAJ) apple juice infused, sliced (SSDC) and whole (WSDC) sucrose infused, and sliced soluble corn fiber, glycerin, sucrose, and sucralose infused (SCFG) (not available whole)) (Table 1). Commercially made 11.34 kg boxes of each treatment were obtained from Ocean Spray Cranberries (Lakeville-Middleboro, MA, USA) within 3 weeks of production from the same plant and repacked into 9.64 g heat sealed transparent bags with oxygen barrier. Treatments were stored for 360 days at 21 °C in a sealed cardboard box from Ocean Spray Cranberries to reduce light exposure. Each of the 5 treatments were stored in their own cardboard box at 21 °C and replicates were removed from the box for each time point. Analytical measurements were taken on the treatments initially (t = 0), weekly for the first month, and monthly until day 360. Analyses were terminated prior to 360 days if two consecutive pulls recorded no quantifiable polyphenols. At each time point, 3 replicates were analyzed for each treatment. Reagents were analytical grade and were purchased from Fisher Scientific (Waltham, MA, USA) unless otherwise noted. 

### 2.1. Proanthocyanidin Content

Replicates were introduced to liquid nitrogen and ground into a powder. The powdered sample (5 g) was placed into a centrifuge tube with 15 mL extraction solution (80% acetone, 19.5% deionized water, 0.5% glacial acetic acid (*v*/*v*)). The test tube was vortexed for 10 s, sonicated in a Branson ultrasonic water bath (Danbury, CT, USA) for 15 min, then centrifuged at 7954× *g* for 20 min. The supernatant was removed from the pellet, the extraction was repeated two more times, and the supernatants combined. After the final extraction each replicate was placed in a glass tube in a Buchi Syncore extraction device (Buchi AG, Flawil, Switzerland) and placed under gradient vacuum at 45 °C for 5 h connected to recirculating chiller set to −10 °C.

Treatments were assessed for proanthocyanidin content using an A2 dimer procyanidin (Indofine Chemical Company, Hillsborough, NJ, USA) standard following a modified method presented by Prior, Fan, Ji, Howell, Nio, Payne, and Reed [18]. For PAC analysis, a Precision XS with 96 well plate (Bio-Tek Instruments, Inc., Winooski, VT, USA) was used for serial dilutions. A2 dimer standard was made by taking 5 mg of procyanidin in a 50 mL volumetric flask and bringing up to volume with ethanol. Using a 96-well plate, 140 µL of blank (80% ethanol in deionized (DI) water), A2 dimer standard, and the replicate were loaded onto the first column for serial dilution conducted by the Precision XS. After dilution, 4-dimethylaminocinnamaldehyde (DMAC) (Sigma-Aldrich, St. Louis, MO, USA) (210 µL) (0.1 DMAC powder in 100 mL 75% ethanol, 12.5% HCl, and 12.5% deionized water (*v*/*v*)) was added to each well. The well plate was loaded into Synergy 2 microplate reader with GEN5 software (Bio-Tek Instruments, Inc., Winooski, VT, USA) and analyzed at 25 °C and 640 nm every min for 30 min. The highest absorbance over 30 min was used (kinetic curve). Calibration curve was generated from the A2 standard used. The concentration of proanthocyanidins in each replicate was determined using a calibration curve (A2 dimer standard absorbance versus A2 dimer concentration) regression line and the below equation where *c* is the concentration of proanthocyanidins in the extraction (g/L), *d* is the dilution factor, *v* is the volume of the extraction fluid after vacuum (mL), and *w* is the weight of the replicate used for extraction (g).

(1) $$\mathrm{PACs}=\frac{cxdxv}{1000xw}$$

### 2.2. Anthocyanin Content

An Agilent 1260 HPLC (Agilent Technologies, Santa Clara, CA, USA) was used for high performance liquid chromatography (HPLC) analysis of anthocyanins according to the method of Brown and Shipley [19]. Replicates were added to liquid nitrogen and ground until a powder was obtained. The powder (1.5 g) was added to 20 mL of 2% HCl:Methanol. The solution was sonicated in a Branson ultrasonic water bath (Danbury, CT, USA) for 15 min, shaken for 30 min, and centrifuged at 2324× *g* for 5 min. The supernatant was removed from the pellet and used in HPLC analysis.

The HPLC was run with two mobile phases (*v*/*v*) consisting of (A) DI water and o-phosphoric acid (99.5:0.5) and (B) DI water, acetonitrile, acetic acid, and o-phosphoric acid (50:48.5:1.0:0.5). Each replicate was analyzed via HPLC for 35 min with a 10 µL injection volume, 1.0 mL/min flow rate, and the absorbance at 520 nm and 25 °C, using a Water X-Select HSS T3 5µm, 4.6 × 150 mm reversed-phase C18 column. Replicates were analyzed for cyanidin-3-galactoside (cy-3-gal), cyanidin-3-glucoside (cy-3-glu), cyanidin-3-arabinoside (cy-3-arab), peonidin-3-galactoside (peo-3-gal), peonidin-3-glucoside (peo-3-glu), and peonidin-3-arabinoside (peo-3-arab). Standards for cyanidin-3-galactoside (cy-3-gal), cyanidin-3-glucoside (cy-3-glu), cyanidin-3-arabinoside (cy-3-arab), and peonidin-3-glucoside (peo-3-glu) were obtained via Phytolab (Vestenbergsgreuth, Germany). Peonidin-3-glucoside (peo-3-glu) and peonidin-3-arabinoside (peo-3-arab) were reported as peonisin-3-glucoside (peo-3-glu) equivalents due to limited commercial manufacturing of the standards and the retention times were based on those validated by Brown and Shipley [19]. The minimum detection limit for cyanidins was 0.02 µg/mL and for peonidins 0.01 µg/mL. Flow rate was consistent (1.0 mL/min) and ratio of mobile phase A to mobile phase B was decreased for the first 32 min and then increased to the initial settings for the remaining 3 min. The anthocyanin content of each sample was calculated using Agilent Open lab software (Agilent Technologies, Santa Clara, CA, USA) based on the extraction dilution and reported as ppm.

### 2.3. Total Phenolic Content

Total phenolic content (TPC) was measured using the Folin–Ciocalteu colorimetric assay [20,21]. Each replicate (10 g) was placed in a Warren blender with 90 g DI water and blended on the low setting for 3 min. Each replicate was analyzed in duplicate and DI water was the blank. Diluted replicates and blank (100 µL) were pipetted into a glass test tube. DI water (3.9 mL) was added to each test tube and vortexed for 5 s. Folin–Ciocalteu reagent (250 µL) (Fischer Scientific, Waltham, MA) was added to the test tube and vortexed for 5 s. Sodium carbonate solution (7.5% sodium carbonate anhydrous (Sigma-Aldrich, St. Louis, MO, USA) in DI water) (750 µL) was added to each test tube and vortexed for 5 s. The replicates were stored in the dark for 30 min and absorbance was read at 765 nm. Gallic acid (Sigma-Aldrich, St. Loius, MO, USA) was used to create a standard calibration curve where 0.5% gallic acid solution was prepared and diluted to 0, 25, 50, 100, 150, and 200 mg/L of gallic acid and the standard curve was made by plotting absorbance vs. concentration. TPC in the replicates were determined by using the gallic acid calibration curve, dilution factor of the replicate, and the moisture content of the replicate to report TPC as mg/g gallic acid equivalent (GAE).

### 2.4. Water Activity and Moisture Content

Water activity (A_w_) was measured using a calibrated Aqua Lab 4TE (Meter Group Inc., Pullman, WA, USA). Each replicate was measured in triplicate and calibrated using LiCl 8.57 molal in H_2_O standard at 0.500 A_w_ (Meter Group Inc., Pullman, WA, USA). Moisture content was assessed via Karl-Fischer titration using a calibrated Metrohm KF 901 Titrando auto-titrator (Metrohm, Herisau, Switzerland). Each replicate (5 g) was added to a stainless-steel homogenization flask and 100 g of Karl-Fischer Grade low water methanol was added. The replicate was then homogenized for 5 min using an Omni Mixer (Omni International) (Kennesaw, GA, USA). After homogenization, the flask was disconnected and covered with parafilm. The liquid sat undisturbed for 5 min before being analyzed for moisture content. Each replicate was then taken into a 3 mL syringe and run through the auto-titrator. Each replicate was analyzed in triplicate.

### 2.5. Colorimetric and Texture Analyses

Color was analyzed using a calibrated handheld CR-410 Colorimeter (Konica Minolta) (Tokyo, Japan). Each replicate was read 5 times by the handheld colorimeter, which was rotated 90° between readings and the average of the readings was reported for L*, a*, and b* values which were used to calculate ΔE according to the below calculation. Each replicate analyzed in triplicate.

(2) $$\Delta E=\sqrt{\left(L_{1}^{*}-L_{2}^{*}{}^{2}\right)+{\left(a_{1}^{*}-a_{2}^{*}\right)}^{2}+{\left(b_{1}^{*}-b_{2}^{*}\right)}^{2}}$$

Texture was analyzed using a calibrated TA.XT.Plus from Texture Technologies Corporation (Hamilton, MA, USA). The TA.XT.Plus was configured with a TA-30 cylinder probe (3” diameter aluminum cylinder, 10mm height), 0.50 mm/s pre-test speed, 0.5 mm/s test speed, 10 mm/s post-test speed, 500 g applied force, 10 mm return distance, and 10 s contact time. Each replicate (25 g) was loaded onto the rounded base plate and the test was run. Adhesion force (g) was recorded by the texture analysis software (Exponent 32, version 6, Texture Technologies Cop, Scarsdale, NY, USA). Each replicate was analyzed in triplicate.

### 2.6. Statistical Analyses

Data were analyzed using Minitab 16 (State College, PA, USA) for analysis of variance (ANOVA) for all treatments. Shapiro–Wilk test for normality was assessed prior to ANOVA. Tukey’s Honest Significant Difference Test (HSD) was used for post-hoc analyses. Significance was set at *p* < 0.05.

## 3. Results and Discussion

### 3.1. Proanthocyanidin Content

All treatments had significant (*p* < 0.05) decline in proanthocyanidin content over time indicating that proanthocyanidins are unstable in SDC regardless of product matrix (Table 2). Initial proanthocyanidin content in SDC was comparable to the values seen by Blumberg et al [5]. Sliced treatments resulted in significantly higher proanthocyanidin content (initial and final) than whole treatments. Slicing of the fruit prior to thermal processing allows for higher infusion rate due to larger infusion surface area which introduces more polyphenols. There was an increase in proanthocyanidin content in the first three time points analyzed for SSDC. This could be due to the natural variation of proanthocyanidin content in fresh cranberries, as the content is not standardized for fresh fruit. SCFG had significantly higher initial and final proanthocyanidin content compared to other sliced treatments, indicating that bulking agents in SCFG such as soluble corn fiber and glycerin may have insulating effects compared to sucrose and apple juice, and thus reduce the degradation of polyphenols. A study on sour cherry puree found that natural sweeteners such as palm sugar, erythritol, xylitol, and other agents such as inulin, inhibited polyphenol degradation, which could be the case in the SCFG treatment [22]. None of the treatments had an initial or final proanthocyanidin content comparable to raw cranberries (3.59 mg/g) [10]. Raw cranberries had a significantly higher initial proanthocyanidin content than all treatments in the study, indicating that regardless of product matrix, thermal processing leads to a significant reduction in proanthocyanidins compared to raw cranberries. Overall degradation of proanthocyanidins (88.3% in SAJ, 87.8% in WAJ, 92.8% in SSDC, 100% in WSDC, and 87.8% in SCFG) indicate that proanthocyanidins are unstable in SDC over time. The continued degradation of proanthocyanidins during storage indicates that while proanthocyanidins do degrade during processing and it is significant compared to raw cranberries, oxidation reactions and polymerization of proanthocyanidins during storage lead to further degradation.

### 3.2. Anthocyanin Content

For all treatments there was a significant (*p* < 0.05) decrease in anthocyanins indicating that anthocyanins significantly degrade over time regardless of product matrix (Table 3). Initial anthocyanin content in SDC was comparable to values reported in Blumberg et al [5]. As previously discussed with proanthocyanidins, slicing resulted in a significantly higher anthocyanin content (both initial and final) compared to whole treatments. Both whole treatments ended the study with no quantifiable anthocyanin content. Whole cranberries are larger individual pieces than the slices cranberries, so it is possible that a combination of higher heat and longer drying time to achieve the same A_w_ causes the significant reduction in anthocyanins. Anthocyanins are susceptible to heat degradation, so increased time in the drier or increased drying temperature in the whole treatments could significantly lower the anthocyanin content compared to the sliced treatments. In a study comparing the anthocyanin content of fresh plums to prunes, high drying temperatures led to degradation of anthocyanins [23] which was mirrored in this study. SCFG had the highest initial anthocyanin (72.61 ppm) while WSDC had the lowest initial anthocyanin content (3.14 ppm). As previously discussed, SCFG bulking agents such as soluble corn fiber and glycerin in may have insulated some of the anthocyanin compounds, resulting in significantly higher initial and final anthocyanin values compared to other sliced treatments [22]. Compared to raw cranberries (604.20 ppm anthocyanins) [9] SDC provide significantly fewer anthocyanins initially, which then degrade over time. Overall degradation of anthocyanins (93.5% SAJ, 100% WAJ, 97.8% SSDC, 100% WSDC, and 98% SCFG) indicate that anthocyanins in SDC are unstable and decrease more than proanthocyanidins. Flavan-3-ol monomer polymerization induced by oxidation via processing produces proanthocyanidins [24], so as monomers are polymerized proanthocyanidins may appear more stable than anthocyanins, which are not formed during this polymerization. While degradation of anthocyanins is a result of drying conditions (time and temperature), further degradation occurs during storage indicating that oxidation reactions occur and degrade anthocyanins further during storage. There was no significant difference in the rates of degradation across treatments, indicating that independent of product matrix, copigmentation in SDC does not provide stability in the lower quantities of anthocyanins displayed in this study. Copigmentation occurs when anthocyanin molecules stack on top of each other providing insulation of compounds from oxidation reactions which may degrade anthocyanins [25,26]. At low levels of anthocyanins seen in the treatments there is not a large enough quantity of anthocyanins for stacking and copigmentation to prevent degradation. 

### 3.3. Total Phenolic Content

For all treatments, there was a significant (*p* < 0.05) difference in the initial TPC, but no significant difference in the final TPC, indicating that TPC stabilizes over time regardless of initial value (Table 4). TPC values were comparable to values reported in Grace et al., 2012 for SDC. Unlike previously discussed with proanthocyanidins and anthocyanins, structure had no impact on the overall TPC in the samples, with WAJ having the highest initial TPC (5.87 mg/g) while WSDC had the lowest initial TPC (2.64 mg/g). WAJ and SAJ contain added apple juice which provides additional polyphenols resulting in higher TPC than SSDC and WSDC. This is not mirrored in anthocyanins and proanthocyanidins, because the major polyphenols in apple juice are in the form of chlorogenic acid, as well as some quercetin [27] which would not be read as anthocyanins or proanthocyanidins. Compared to raw cranberries which were tested to have 1.60 mg/g TPC, all matrices of SDC had significantly higher initial TPC as well as final TPC. Treatments with the highest TPC compared to the raw cranberries were WAJ, SCFG, and SAJ. The WAJ and SAJ both are composed of additional apple juice concentrate containing quercetin, which in studies have been showed to retain TPC and can be regenerated upon heating to various quercetin glycosides which may result in the higher TPC [15,28,29]. Compared to anthocyanins and proanthocyanidins, TPC are significantly more stable, only decreasing by 42.7% in SAJ, 56.9% in WAJ, 13.9% in SSDC, 10.9% in WSDC, and 51.6% in SCFG. Proanthocyanidins and anthocyanins are only two compounds that make up the overall total phenolic content in cranberries, so while anthocyanins and proanthocyanidins saw more degradation throughout shelf-life, the rest of the TPC such as acids and other flavonols are more stable—potentially leading to more stability in overall TPC. SSDC and WSDC had the lowest initial PAC content (2.69 mg/g for SSDC and 2.64 mg/g for WSDC, respectively), but there was no significant decrease in TPC for these treatments throughout shelf life. SAJ, WAJ, and SCFG saw significant decreases in their TPC due to higher initial content which eventually decreased to the content initially seen in SSDC and WSDC. 

### 3.4. Water Activity and Moisture Content

For all treatments, A_w_ did not significantly (*p* > 0.05) change over time, indicating that A_w_ is stable in SDC regardless of product matrix (data not shown). A_w_ refers to the unbound or free water in a food system whereas moisture content refers to the total amount of water in a food system. SSDC was the only treatment without a significant increase in moisture content between initial (14.37%) and final (14.70%), indicating that while A_w_ does not significantly change, SDC are more susceptible to changes in the total amount of water in the matrix. Structure does not appear to significantly impact moisture content over time, as WAJ decreased in moisture content (16.70% to 14.82%) while WSDC increased in moisture content (13.40% to 15.65%) and SAJ decreased in moisture content (16.52% to 15.28%) while there was no significant change in SSDC. SCFG had the highest initial moisture content (18.42%), mirroring the largest A_w_. Glycerin has been shown to have moisture absorption properties which would help in the drying process but has also been shown to have poor moisture retention which would cause the water activity to be higher in SCFG [30]. SSDC and WSDC had a significantly lower initial moisture content (14.37% and 13.40%, respectively). As previously seen with A_w_, sucrose has a lower effect on glass transition temperature, so sucrose-infused treatments (SSDC and WSDC) also have a lower moisture content [31]. This is mirrored in A_w_, indicating that sucrose infusion in the cranberries pre-drying also resulted in a lower amount of free water in SSDC and WSDC treatments. While final A_w_ between treatments differed, differences in initial moisture content did not cause significant differences in final moisture content between treatments. A study with dried apricots demonstrated that diffusivity in dried apricots during storage was affected by the glass transition (as previously seen with A_w_) resulting in a decrease in moisture content over time [32]. This effect is seen in all SDC treatments except for WSDC, which increased in moisture—possibly because of the whole structure resulting in more free moisture.

### 3.5. Colorimetric and Texture Analyses

#### 3.5.1. Color

There was a significant (*p* < 0.05) increase in DE over time, indicating color degradation in all treatments over time (Table 5). Studies have found that blueberries and strawberries undergo enzymatic browning after food processing which deteriorates color [33,34]. Polyphenol oxidase (PPO) is a naturally occurring enzyme that causes browning in the presence of oxygen when the phenolase reacts with oxygen causing phenols to convert to melanin (brown pigment) [33,35,36]. PPO can degrade anthocyanins to brown, black, or yellow colors from the natural red, purple, and blue pigments [34]. Processing such as juicing and drying as well as storage conditions can cause PPO reactions causing most color changes and anthocyanin polymerization reactions [33,34,37]. Color change was not influenced by slicing, as SCFG had the largest DE (6.75) but WAJ had the second largest DE (4.28). SCFG had the largest change in color, which could be due to the significantly higher amount of anthocyanins and proanthocyanidins in that treatment available for PPO reactions to occur. WAJ also had the largest TPC, which could explain why it was higher in DE. Aside from PPO reactions, other browning and color deterioration may occur due to the Maillard reaction, caramelization of sugars during processing and storage, and overall pigment deterioration [38,39].

#### 3.5.2. Texture

Treatments of similar product matrices (SAJ/WAJ and SSDC/WSDC) did not have significantly (*p* > 0.05) different textures, indicating that slicing does not have an impact on texture compared to whole (Table 6). SCFG had significantly higher adhesion force initially, indicating that SCFG was significantly more sticky than other treatments. SCFG had the highest initial moisture content and A_w_ among the treatments, which would cause excess free and total water in the sample, leading to more adhesion. Studies of pear fruit leather found, similarly, that additional water (moisture content or A_w_) increased cohesion and surface moisture/adhesion [40]. The same pear fruit leather study also found that both corn syrup and pectin in pear fruit leather increased adhesion, which could be seen similarly here with the added bulking ingredients in SCFG [40]. Adhesion force decreasing is an indication of the treatments drying out during shelf life. SAJ, WAJ, and SSDC retained their initial adhesion properties while WSDC became more adhesive, and SCFG became less adhesive and drier. For WSDC and SCFG this change in adhesion was mirrored with the increase (WSDC) and decrease (SCFG) in moisture content. WSDC increased significantly in moisture content throughout shelf life, becoming stickier and thus increasing in adhesion properties. SCFG conversely decreased significantly in moisture content, leading to decreased adhesion properties and becoming drier. A_w_ properties also were mirrored in adhesion properties, with SSDC and WSDC having the lowest initial A_w_ and moisture content and therefore having the lowest adhesion force. Across treatments, apple juice infused treatments were significantly more adhesive than sucrose infused treatments, but less adhesive than SCFG, mirroring the results found in moisture content and A_w_. 

## 4. Conclusions

All treatments of SDC had a significant reduction in anthocyanins and proanthocyanidins, and those treatments with larger TPC initially also showed a significant reduction in TPC compared to those treatments with lower initial TPC. Sliced treatments had higher initial polyphenols (proanthocyanidins, anthocyanins, TPC) than the whole treatments due to the increased infusion of polyphenols within the sliced format. While treatments did not show a significant change in A_w_, change in moisture content varied between treatments. While sweetened dried cranberries are a convenient option for a snack, compared to raw cranberries, the bioactive compounds are negatively impacted by processing limiting application as a functional food. Processing and storage parameters could be optimized to minimize polyphenol degradation and better position SDC as functional foods.

Examples include: (1) reduce the time and temperature of the drying process to better retain the bioactive compounds found in fresh cranberries, (2) introduce a nitrogen flush to the packaging to limit oxygen exposure and oxidation reactions during storage, and (3) utilize different packaging materials which may be better inhibitors of oxidation.

## Figures and Tables

**Table 1:** Overview of Sweetened Dried Cranberries Evaluated in Study.

Treatment	Sliced Apple Juice Infused	Whole Apple Juice Infused	Sliced Sucrose Infused	Whole Sucrose Infused	Sliced Soluble Corn Fiber, Glycerin, Sucrose, and Sucralose Infused
**Acronym**	SAJ	WAJ	SSDC	WSDC	SCFG
**Structure**	Sliced	Whole	Sliced	Whole	Sliced
**Ingredients**	Cranberries, Apple Juice Concentrate	Cranberries, Apple Juice Concentrate	Cranberries, Sugar	Cranberries, Sugar	Cranberries, Soluble Corn Fiber, Sugar, Glycerin, Sucralose
**Initial Water Activity (A_w)_**	0.5360 ^b^	0.5415 ^b^	0.4831 ^c^	0.4953 ^c^	0.5974 ^a^
**Initial Moisture (%)**	16.86 ^a^	16.70 ^a^	14.37 ^b^	13.40 ^b^	18.42 ^a^

Values not sharing the same letter are significantly different (*p* < 0.05) within rows analyzed by ANOVA (Tukey’s HSD Test). A_w_ and moisture content values indicated are initial values for each treatment.

**Table 2:** Proanthocyanidin Content (mg/g) of Sweetened Dried Cranberries over Time.

Time (Days)	0	7	14	21	28	60	90	120	150	180	210	240	270	300	330	360
Sliced Apple Juice Infused (SAJ)	0.56 ^aB^ ± 0.07	0.48 ^aC^ ± 0.06	0.51 ^aB^ ± 0.08	0.44 ^abB^ ± 0.12	0.41 ^abB^ ± 0.10	0.31 ^bcB^ ± 0.05	0.24 ^cdB^ ± 0.03	0.28 ^bcdA^ ± 0.03	0.24 ^cdA^ ± 0.02	0.21 ^cdeA^ ± 0.01	0.22 ^cdeA^ ± 0.04	0.21 ^cdeA^ ± 0.00	0.20 ^cdeA^ ± 0.02	0.20 ^cdeA^ ± 0.02	0.11 ^deA^ ± 0.02	0.07 ^eB^ ± 0.01
Whole Apple Juice Infused (WAJ)	0.30 ^bC^ ± 0.06	0.41 ^aC^ ± 0.07	0.29 ^bC^ ± 0.04	0.25 ^bcC^ ± 0.01	0.26 ^bcC^ ± 0.04	0.18 ^cdC^ ± 0.03	0.13 ^deC^ ± 0.02	0.14 ^deB^ ± 0.02	0.09 ^defB^ ± 0.01	0.13 ^deB^ ± 0.03	0.13 ^deB^ ± 0.00	0.09 ^efB^ ± 0.00	0.06 ^efB^ ± 0.01	0.03 ^fB^ ± 0.01	0.02 ^fC^ ± 0.00	0.04 ^fC^ ± 0.00
Sliced Sucrose Infused (SSDC)	0.61 ^cB^ ± 0.12	0.89 ^bA^ ± 0.03	0.90 ^bA^ ± 0.03	1.19 ^aA^ ± 0.09	0.96 ^bA^ ± 0.04	0.50 ^cdA^ ± 0.05	0.44 ^deA^ ± 0.04	0.33 ^efA^ ± 0.03	0.22 ^fgA^ ± 0.01	0.19 ^fghA^ ± 0.02	0.20 ^fghA^ ± 0.02	0.18 ^ghiA^ ± 0.02	0.09 ^ghiB^ ± 0.01	0.09 ^ghiB^ ± 0.01	0.08 ^hiAB^ ± 0.01	0.04 ^iC^ ± 0.01
Whole Sucrose Infused (WSDC)	0.84 ^aA^ ± 0.08	0.62 ^bB^ ± 0.04	0.53 ^bB^ ± 0.05	0.33 ^cC^ ± 0.04	0.22 ^dC^ ± 0.03	0.11 ^eC^ ± 0.03	0.07 ^efC^ ± 0.02	0.03 ^efC^ ± 0.01	0.05 ^efB^ ± 0.03	0.05 ^efC^ ± 0.01	0.03 ^efC^ ± 0.00	0.02 ^fB^ ± 0.00	0.00 ^fB^ ± 0.00	0.00 ^fC^ ± 0.00	Not Performed	Not Performed
Sliced Soluble Corn Fiber, Glycerin, Sucrose, and Sucralose Infused (SCFG)	1.04 ^aA^ ± 0.12	0.59 ^bB^ ± 0.08	0.53 ^bcB^ ± 0.05	0.55 ^bcB^ ± 0.04	0.44 ^cdB^ ± 0.01	0.31 ^deB^ ± 0.01	0.24 ^efB^ ± 0.02	0.27 ^eA^ ± 0.02	0.24 ^efA^ ± 0.01	0.21 ^efA^ ± 0.01	0.23 ^efA^ ± 0.05	0.21 ^efA^ ± 0.01	0.21 ^efA^ ± 0.02	0.23 ^efA^ ± 0.02	0.12 ^fA^ ± 0.01	0.13 ^fA^ ± 0.01

Proanthocyanidin content values were recorded as mg/g. Each value is the average ± standard deviation (*n* = 3). Treatments were stored at 21 °C in individually sealed bags in boxes to limit light exposure before analytical measurements. Values within rows not sharing a lowercase letter are significantly (*p* < 0.05) different. Values within columns not sharing an uppercase letter are significantly (*p* < 0.05) different. Treatments were analyzed by ANOVA (Tukey’s HSD).

**Table 3:** Anthocyanin Content (ppm) of Sweetened Dried Cranberries over Time.

Time (Days)	0	7	14	21	28	60	90	120	150	180	210	240	270	300	330	360
Sliced Apple Juice Infused (SAJ)	22.72 ^aC^ ± 0.20	16.75 ^bB^ ± 1.86	16.58 ^bB^ ± 0.90	12.21 ^cB^ ± 1.84	13.61 ^cB^ ± 0.09	8.70 ^dB^ ± 0.69	6.03 ^eB^ ± 0.47	4.70 ^efC^ ± 0.64	3.90 ^efgB^ ± 0.16	3.22 ^fghB^ ± 0.13	3.51 ^fghB^ ± 0.29	2.70 ^fghB^ ± 0.17	2.59 ^fghB^ ± 0.11	1.93 ^ghA^ ± 0.22	1.52 ^hA^ ± 0.14	1.47 ^hA^ ± 0.28
Whole Apple Juice Infused (WAJ)	12.42 ^aD^ ± 0.36	8.19 ^bC^ ± 0.94	6.53 ^cC^ ± 0.71	6.31 ^cBC^ ± 0.66	4.77 ^dC^ ± 0.95	3.14 ^eC^ ± 0.15	2.26 ^efC^ ± 0.16	1.44 ^fgD^ ± 0.09	1.10 ^fghC^ ± 0.04	0.87 ^ghC^ ± 0.38	0.78 ^ghD^ ± 0.02	0.36 ^ghC^ ± 0.15	0.08 ^hD^ ± 0.14	0.08 ^hB^ ± 0.14	0.00 ^hB^ ± 0.00	0.00 ^hB^ ± 0.00
Sliced Sucrose Infused (SSDC)	40.10 ^aB^ ± 1.00	41.35 ^aA^ ± 2.54	37.98 ^abA^ ± 3.07	33.56 ^bA^ ± 1.01	28.73 ^cA^ ± 2.98	27.33 ^cA^ ± 2.62	10.68 ^dA^ ± 0.75	9.31 ^deA^ ± 0.97	5.32 ^efA^ ± 0.73	4.93 ^efA^ ± 0.13	4.45 ^fA^ ± 0.12	4.57 ^fA^ ± 0.18	3.59 ^fA^ ± 0.31	2.07 ^fA^ ± 0.08	1.61 ^fA^ ± 0.27	0.89 ^fAB^ ± 0.23
Whole Sucrose Infused (WSDC)	3.14 ^aE^ ± 0.10	3.02 ^aD^ ± 0.03	2.38 ^bD^ ± 0.26	2.09 ^bC^ ± 0.06	1.55 ^cD^ ± 0.07	1.06 ^dD^ ± 0.05	0.67 ^eD^ ± 0.07	0.30 ^fE^ ± 0.31	0.08 ^fD^ ± 0.14	0.00 ^fD^ ± 0.00	0.00 ^fE^ ± 0.00	Not Performed	Not Performed	Not Performed	Not Performed	Not Performed
Sliced Soluble Corn Fiber, Glycerin, Sucrose, and Sucralose Infused (SCFG)	72.61 ^aA^ ± 2.50	40.25 ^bA^ ± 1.43	23.07 ^cB^ ± 7.61	22.31 ^cA^ ± 1.00	16.88 ^cB^ ± 1.94	8.33 ^dB^ ± 1.44	6.20 ^deB^ ± 0.63	6.71 ^deB^ ± 0.21	3.48 ^deB^ ± 0.47	3.17 ^deB^ ± 0.33	2.30 ^deC^ ± 0.43	1.97 ^deB^ ± 0.33	1.83 ^deC^ ± 0.17	1.57 ^eA^ ± 0.10	1.46 ^eA^ ± 0.56	1.45 ^eA^ ± 0.61

Anthocyanin content values were recorded as ppm. Each value is the average ± standard deviation (*n* = 3). Treatments were stored at 21 °C in individually sealed bags in boxes to limit light exposure before analytical measurements. Values within rows not sharing a lowercase letter are significantly (*p* < 0.05) different. Values within columns not sharing an uppercase letter are significantly (*p* < 0.05) different. Treatments were analyzed by ANOVA (Tukey’s HSD).

**Table 4:** Total Phenolic Content (mg/g) of Sweetened Dried Cranberries over Time.

Time (Days)	0	7	14	21	28	60	90	120	150	180	210	240	270	300	330	360
Sliced Apple Juice Infused (SAJ)	4.37 ^aC^ ± 0.20	4.51 ^abcB^ ± 0.25	4.59 ^abC^ ± 0.08	4.28 ^abcC^ ± 0.11	4.51 ^abcB^ ± 0.08	3.84 ^cC^ ± 0.11	3.99 ^bc^ ± 0.07	2.85 ^dA^ ± 0.33	2.57 ^dA^ ± 0.02	2.58 ^dA^ ± 0.04	2.56 ^dA^ ± 0.07	2.56 ^dA^ ± 0.05	2.51 ^dA^ ± 0.06	2.55 ^dA^ ± 0.09	2.61 ^dA^ ± 0.17	2.51 ^dA^ ± 0.18
Whole Apple Juice Infused (WAJ)	5.87 ^aA^ ± 0.46	5.34 ^abA^ ± 0.09	5.42 ^abA^ ± 0.15	5.27 ^bA^ ± 0.06	5.58 ^abA^ ± 0.20	5.36 ^abA^ ± 0.24	3.29 ^c^ ± 0.32	2.75 ^cdA^ ± 0.08	2.65 ^dA^ ± 0.08	2.43 ^dA^ ± 0.05	2.80 ^cdA^ ± 0.11	2.79 ^cdA^ ± 0.07	2.94 ^cdA^ ± 0.22	2.94 ^cdA^ ± 0.05	2.64 ^dA^ ± 0.10	2.53 ^dA^ ± 0.04
Sliced Sucrose Infused (SSDC)	2.69 ^aD^ ± 0.06	2.60 ^abcC^ ± 0.02	2.50 ^abcDde^ ± 0.02	2.61 ^abD^ ± 0.04	2.54 ^abcCd^ ± 0.11	2.53 ^abcdD^ ± 0.09	2.66 ^ab^ ± 0.13	2.28 ^deA^ ± 0.09	2.22 ^eA^ ± 0.08	2.26 ^deA^ ± 0.09	2.24 ^eA^ ± 0.07	2.29 ^deA^ ± 0.08	2.38 ^bcdeA^ ± 0.03	2.24 ^eA^ ± 0.11	2.26 ^deA^ ± 0.23	2.32 ^cdeA^ ± 0.05
Whole Sucrose Infused (WSDC)	2.64 ^aD^ ± 0.08	2.62 ^aC^ ± 0.11	2.59 ^aD^ ± 0.07	2.49 ^abD^ ± 0.02	2.44 ^abcC^ ± 0.04	2.56 ^aD^ ± 0.10	2.55 ^ab^ ± 0.03	2.40 ^abcAd^ ± 0.09	2.27 ^bcdAe^ ± 0.06	2.09 ^deA^ ± 0.05	2.23 ^bcdAe^ ± 0.20	2.40 ^abcdA^ ± 0.18	2.43 ^abcA^ ± 0.08	2.16 ^cdeA^ ± 0.08	2.05 ^eA^ ± 0.08	2.35 ^abcdeA^ ± 0.17
Sliced Soluble Corn Fiber, Glycerin, Sucrose, and Sucralose Infused (SCFG)	5.04 ^aB^ ± 0.03	4.35 ^bcdB^ ± 0.54	4.95 ^aB^ ± 0.03	4.67 ^abcB^ ± 0.17	4.85 ^abB^ ± 0.12	4.04 ^dB^ ± 0.08	4.28 ^cd^ ± 0.11	2.87 ^eA^ ± 0.43	2.33 ^eA^ ± 0.05	2.37 ^eA^ ± 0.01	2.46 ^eA^ ± 0.02	2.47 ^eA^ ± 0.03	2.46 ^eA^ ± 0.03	2.41 ^eA^ ± 0.05	2.58 ^eA^ ± 0.15	2.44 ^eA^ ± 0.02

Total phenolic content values were recorded as mg/g. Each value is the average ± standard deviation (*n* = 3). Treatments were stored at 21 °C in individually sealed bags in boxes to limit light exposure before analytical measurements. Values within rows not sharing a lowercase letter are significantly (*p* < 0.05) different. Values within columns not sharing an uppercase letter are significantly (*p* < 0.05) different. Treatments were analyzed by ANOVA (Tukey’s HSD).

**Table 5:** DE of Sweetened Dried Cranberries over Time.

Time (Days)	0	7	14	21	28	60	90	120	150	180	210	240	270	300	330	360
Sliced Apple Juice Infused (SAJ)	0.00 ^fA^ ± 0.00	1.49 ^efB^ ± 0.96	1.28 ^efB^ ± 0.99	2.18 ^defA^ ± 0.99	1.41 ^efAB^ ± 0.34	1.44 ^efB^ ± 0.28	3.59 ^abcdeB^ ± 0.73	4.95 ^abcA^ ± 1.80	4.16 ^abcdB^ ± 0.60	5.58 ^abA^ ± 1.47	6.11 ^aA^ ± 0.45	5.81 ^abB^ ± 1.01	5.75 ^abB^ ± 0.43	3.59 ^abcdeB^ ± 0.35	2.75 ^cdeC^ ± 0.76	3.39 ^bcdeBC^ ± 0.33
Whole Apple Juice Infused (WAJ)	0.00 ^fA^ ± 0.00	0.71 ^efC^ ± 0.01	2.18 ^cdA^ ± 0.39	3.47 ^abA^ ± 0.41	1.98 ^deA^ ± 0.72	2.96 ^bcdA^ ± 0.27	3.67 ^abB^ ± 0.85	3.22 ^abB^ ± 0.32	3.47 ^abC^ ± 0.29	3.56 ^abC^ ± 0.04	3.47 ^abC^ ± 0.12	3.89 ^abC^ ± 0.26	3.52 ^abC^ ± 0.21	3.70 ^abB^ ± 0.08	3.96 ^abB^ ± 0.16	4.28 ^aB^ ± 0.56
Sliced Sucrose Infused (SSDC)	0.00 ^hA^ ± 0.00	0.76 ^fghC^ ± 0.21	0.85 ^fghC^ ± 0.10	0.46 ^ghC^ ± 0.03	0.53 ^fghC^ ± 0.10	1.71 ^efgB^ ± 0.36	1.75 ^efC^ ± 0.17	3.93 ^abcB^ ± 0.22	4.99 ^aB^ ± 0.84	4.18 ^abcB^ ± 0.22	4.82 ^abB^ ± 0.45	3.66 ^bcdC^ ± 0.51	4.10 ^abcC^ ± 0.87	3.74 ^abcB^ ± 0.36	3.33 ^cdB^ ± 0.57	2.42 ^deC^ ± 0.43
Whole Sucrose Infused (WSDC)	0.00 ^hA^ ± 0.00	0.68 ^ghC^ ± 0.08	0.87 ^fghC^ ± 0.20	0.99 ^efghB^ ± 0.51	1.18 ^defghB^ ± 0.07	1.28 ^defgB^ ± 0.14	2.03 ^bcdefC^ ± 0.61	2.16 ^bcdeC^ ± 0.52	3.12 ^abC^ ± 0.49	3.45 ^aC^ ± 0.35	3.17 ^abC^ ± 0.60	2.19 ^bcdeD^ ± 0.20	1.84 ^cdefgD^ ± 0.24	2.25 ^abcdC^ ± 0.42	2.65 ^abcC^ ± 0.42	2.50 ^abcC^ ± 0.70
Sliced Soluble Corn Fiber, Glycerin, Sucrose, and Sucralose Infused (SCFG)	0.00 ^dA^ ± 0.00	2.33 ^cA^ ± 1.19	1.62 ^cdAB^ ± 0.50	2.71 ^cA^ ± 1.09	2.37 ^cA^ ± 0.23	3.39 ^bcA^ ± 0.50	5.19 ^abA^ ± 0.92	5.83 ^aA^ ± 0.79	6.52 ^aA^ ± 0.50	5.82 ^aA^ ± 0.55	6.75 ^aA^ ± 0.23	6.96 ^aA^ ± 0.37	7.18 ^aA^ ± 1.42	6.46 ^aA^ ± 0.23	6.23 ^aA^ ± 0.48	6.75 ^aA^ ± 0.12

Each value is the average ± standard deviation (*n* = 3). Treatments were stored at 21 °C in individually sealed bags in boxes to limit light exposure before analytical measurements. Values within rows not sharing a lowercase letter are significantly (*p* < 0.05) different. Values within columns not sharing an uppercase letter are significantly (*p* < 0.05) different. Treatments were analyzed by ANOVA (Tukey’s HSD).

**Table 6:** Adhesion Force (g) of Sweetened Dried Cranberries over Time.

Time (Days)	0	7	14	21	28	60	90	120	150	180	210	240	270	300	330	360
Sliced Apple Juice Infused (SAJ)	199.67 ^aB^ ± 58.03	130.73 ^aA^ ± 3.96	172.57 ^aA^ ± 48.97	198.17 ^aA^ ± 16.91	156.10 ^aA^ ± 7.85	140.50 ^aA^ ± 19.66	183.00 ^aA^ ± 14.63	174.00 ^aA^ ± 36.29	164.31 ^aA^ ± 52.39	161.33 ^aA^ ± 43.57	220.61 ^aA^ ± 34.49	169.34 ^aA^ ± 7.98	204.32 ^aA^ ± 26.25	194.92 ^aA^ ± 21.54	216.74 ^aA^ ± 30.68	214.88 ^aA^ ± 21.06
Whole Apple Juice Infused (WAJ)	180.07 ^aB^ ± 27.03	164.23 ^abA^ ± 28.93	93.90 ^bB^ ± 54.14	104.67 ^abB^ ± 20.55	96.35 ^bB^ ± 16.10	140.13 ^abA^ ± 14.77	127.22 ^abB^ ± 39.75	154.71 ^abA^ ± 27.55	116.72 ^abB^ ± 31.31	102.33 ^abB^ ± 15.75	130.54 ^abC^ ± 25.17	103.09 ^abB^ ± 15.85	143.56 ^abA^ ± 7.10	141.61 ^abA^ ± 26.02	155.31 ^abA^ ± 16.41	176.79 ^aA^ ± 13.89
Sliced Sucrose Infused (SSDC)	90.00 ^abcdC^ ± 15.54	126.48 ^aA^ ± 27.91	91.83 ^aBbcd^ ± 22.39	67.59 ^bcdC^ ± 11.02	97.77 ^abcdB^ ± 16.48	75.24 ^bcdB^ ± 11.20	75.16 ^bcdC^ ± 16.83	108.93 ^abcB^ ± 14.16	77.86 ^bcdC^ ± 10.35	78.94 ^abcdC^ ± 13.66	55.37 ^dD^ ± 5.33	64.76 ^cdC^ ± 7.30	73.20 ^bcdB^ ± 16.15	95.66 ^abcdB^ ± 17.16	114.14 ^abB^ ± 17.23	111.90 ^abcB^ ± 19.97
Whole Sucrose Infused (WSDC)	61.25 ^abC^ ± 7.87	76.34 ^abB^ ± 9.56	78.05 ^abC^ ± 12.55	87.22 ^abBC^ ± 15.44	70.68 ^abB^ ± 12.33	71.43 ^abB^ ± 26.21	74.51 ^abC^ ± 21.70	77.85 ^abC^ ± 19.42	73.81 ^abC^ ± 15.45	49.11 ^bD^ ± 16.24	43.03 ^bD^ ± 10.15	54.99 ^abC^ ± 13.84	51.92 ^abB^ ± 15.24	63.43 ^abC^ ± 16.41	85.46 ^abB^ ± 9.30	96.70 ^aB^ ± 16.03
Sliced Soluble Corn Fiber, Glycerin, Sucrose, and Sucralose Infused (SCFG)	292.47 ^aA^ ± 29.88	129.83 ^bA^ ± 9.94	193.20 ^bA^ ± 47.50	156.83 ^bA^ ± 44.43	171.97 ^bA^ ± 28.10	175.95 ^bA^ ± 30.77	185.30 ^bA^ ± 21.90	168.25 ^bA^ ± 2.05	156.28 ^bA^ ± 83.98	182.30 ^bA^ ± 11.35	167.25 ^bB^ ± 22.92	150.02 ^bA^ ± 20.88	185.52 ^bA^ ± 13.60	193.50 ^bA^ ± 8.12	189.12 ^bA^ ± 12.05	184.97 ^bA^ ± 13.77

Adhesion force values were recorded on a g basis. Each value is the average ± standard deviation (*n* = 3). Treatments were stored at 21 °C in individually sealed bags in boxes to limit light exposure before analytical measurements. Values within rows not sharing a lowercase letter are significantly (*p* < 0.05) different. Values within columns not sharing an uppercase letter are significantly (*p* < 0.05) different. Treatments were analyzed by ANOVA (Tukey’s HSD).
